# Supplementary material for: Automating the search for a patent’s prior art with a full text similarity search
Source: PLoS One. 2019 Mar 4;14(3):e0212103. doi: 10.1371/journal.pone.0212103 (PMC6398827; doi:10.1371/journal.pone.0212103)
Supplement: S1 File — A Methods: A1 Feature representations of text documents, A2 Functions for measuring similarity between text documents. B Data. C Evaluation. D Results: D1 Identifying cited patents using different similarity functions with BOW features, D2 Detailed examination of outliers in the citation process. (PDF) [file pone.0212103.s001.pdf]

Automating the search for a patent's prior art with a full text similarity search
SUPPORTING INFORMATION

Lea Helmers[1 *] **Franziska Horn**[1 *] **Franziska Biegler**[2] **Tim Oppermann**[2] **Klaus-Robert Müller**[1,3,4]
[1]Machine Learning Group, Technische Universität Berlin, Berlin, Germany
[2]Pfenning, Meinig & Partner mbB, Berlin, Germany
[3]Department of Brain and Cognitive Engineering, Korea University, Seoul, Republic of Korea
[4]Max-Planck-Institut für Informatik, Saarbrücken, Germany
franziska.horn@campus.tu-berlin.de, klaus-robert.mueller@tu-berlin.de

Supporting information A: Methods

A1 Feature representations of text documents

TF-IDF BOW FEATURES

Given D documents with a vocabulary of size L, each text is transformed into a *bag-of-words* (BOW) feature vector $\mathbf{x}_k \in \mathbb{R}^L\ \forall k \in 1...D$ by first computing a normalized count, the *term frequency* (tf), for each word in a text, and then weighting this by the word's *inverse document frequency* (idf) to reduce the influence of very frequent but inexpressive words that occur in almost all documents (such as 'and' and 'the') [1]. The idf of a term w is calculated as the logarithm of the total number of documents, $|D|$, divided by the number of documents that contain term w, i.e.

$$\text{idf}\,(w) = \log \frac{|D|}{|\{d \in D : w \in d\}|}.$$

The entry corresponding to the word w in the feature vector $\mathbf{x}_k$ of a document k is then

$$\mathbf{x}_k(w) = \text{tf}_k(w) \cdot \text{idf}\,(w).$$

Instead of using the term frequency, a binary entry in the feature vector for each word occurring in the text might often suffice. Furthermore, the final *tf-idf* vectors can be normalized by dividing them e.g. by the maximum or the length of the respective vector:

$$\tilde{\mathbf{x}}_k = \frac{\mathbf{x}_k}{\max_w \mathbf{x}_k(w)} \quad \text{or} \quad \tilde{\mathbf{x}}_k = \frac{\mathbf{x}_k}{\|\mathbf{x}_k\|}.$$

LSA AND KPCA

Transforming the documents in the corpus into BOW vectors leads to a high-dimensional but sparse feature matrix. These feature representations can be reduced to their most expressive dimensions, which helps to reduce noise in the data and create more overlap between vectors. For this, we experiment with both *latent semantic analysis* (LSA) [2] and *kernel principal component analysis* (KPCA) [3].

LSA represents a word's meaning as the average of all the passages the word appears in, and a passage, such as a document, as the average of all the words it contains. Mathematically, a singular value decomposition (SVD) of the BOW feature matrix $X \in \mathbb{R}^{D\times L}$ for the respective corpus is performed. The original data points can then be projected onto the vectors corresponding to the l largest singular values of matrix X, yielding a lower-dimensional representation $\hat{X} \in \mathbb{R}^{D\times l}$, where $l < L$. Choosing a dimensionality l that is smaller than the original dimension L is assumed to lead to a deeper abstraction of words and word sequences and to give a better approximation of their meaning [2].

Similarly, KPCA [3,4] performs an SVD of a linear or non-linear kernel matrix $K \in \mathbb{R}^{D\times D}$ to obtain a low dimensional representation of the data, again based on the eigenvectors corresponding to the largest eigenvalues of this matrix. While

*Equal contribution.

we have studied different Gaussian kernels, we found that good results could already be obtained using the linear kernel $K = XX^\top$.

When reducing the dimensionality of the BOW feature vectors with LSA and KPCA, four embedding dimensions (100, 250, 500 and 1000) were tested and the best performance on the full texts was achieved using 1000 dimensions. As the dataset subsample contains only 450 patent pairs, here the best results with LSA and KPCA were achieved using only 100 dimensions.

Combining BOW features with word2vec embeddings

One shortcoming of the BOW vectors is that semantic relationships between words, such as synonymy, as well as word order, are not taken into account. This is due to the fact that each word is associated with a single dimension in the feature vector and therefore the distances between all words are equal. The aspect of synonymy is especially relevant for patent texts, where very abstract and general terms are used for describing an invention in order to assure a maximum degree of coverage. For instance, a term like *fastener* might be preferred over the usage of the term *screw*, as it includes a wider range of material and therefore gives a better protection against infringement. Thus, patent texts tend to contain neologisms and abstract words that might even be unique in the corpus. To account for this variety in a keyword search is especially tedious and prone to errors as the examiner has to search for synonyms at different levels of abstraction or rely on a thesaurus, which would then need to be kept up-to-date [5]. Even the BOW approach could in this case only capture the similarity between the patent texts if there is overlap between the words in the context around a synonym. An approach specifically developed to overcome these restrictions are *neural network language models* (NNLM) [6], which aim at representing words or documents by semantically meaningful vectorial embeddings.

A NNLM that recently received a lot of attention is *word2vec*. Its purpose is to embed words in a vector space based on their contexts, such that terms appearing in similar contexts are close to each other in the embedding space w.r.t. the cosine similarity [7–9]. Given a text corpus, the word representations are obtained by training a neural network that learns from the local contexts of the input words in the corpus. The embedding is then given by the learned weight matrix. Mikolov et al. [7] describe two different network architectures for training the *word2vec* model, namely the *continuous bag-of-words* (CBOW) and the *skip-gram* model. The first one learns word representations by predicting a target word based on its context words and the latter one by predicting the context words for the current input word. As the skip-gram model showed better performance in analogy tasks [7, 8, 10] it is used in this paper.[1]

To make use of the information learned by the *word2vec* model for each word in the corpus vocabulary L, the trained word embeddings have to be combined to create a document vector for each patent text. To this end, the dot product of each document's BOW vector with the word embedding matrix $W \in \mathbb{R}^{L\times r}$, containing one r-dimensional word embedding per row, is calculated. For each document represented by a BOW vector $\mathbf{x}_k \in \mathbb{R}^L$, this results in a new document vector $\tilde{\mathbf{x}}_k \in \mathbb{R}^r$, which corresponds to the sum of the *word2vec* embeddings of the terms occurring in the document, weighted by their respective *tf-idf* scores. Combining the BOW vectors and the word embeddings thus comes along with a dimensionality reduction of the document vectors, while their sparseness is lost.

For the *word2vec* model we use a standard setting from the literature (i.e. the embedding dimension r was set to 200, the window size c as well as the minimum frequency to 5 and negative sampling was performed using 13 noise words) [7, 8].

Doc2vec representations

With *doc2vec*, Le and Mikolov [11] extend the *word2vec* model to directly represent word sequences of arbitrary lengths, such as sentences, paragraphs or even whole documents, by vectors. To learn the representations, word and paragraph vectors are trained simultaneously for predicting the next word for different contexts of fixed size sampled from the paragraph such that, at least in small contexts, word order is taken into account. Words are mapped to a unique embedding in a matrix $W \in \mathbb{R}^{L\times r}$ and paragraphs to a unique embedding in a matrix $P \in \mathbb{R}^{D\times r}$. In each training step, paragraph and word embeddings are combined by concatenation to predict the next word given a context sampled from the respective paragraph. After training, the *doc2vec* model can be used to infer the embedding for an unseen document by performing gradient descent on the document matrix P after having added more rows to it and holding the learned word embeddings and softmax weights fixed [11].

For the *doc2vec* model, we explored the parameter values 50, 100, 200 and 500 for the embedding dimension r of the document vectors on the cited/random dataset in preliminary experiments, with the best results achieved with $r = 50$. The window size was set to 8, the minimum word count to 5, and the model was trained for 18 iterations. When training the model, the target patents were excluded from the corpus to avoid overfitting. Their document vectors were then inferred by

[1] Analogy tasks aim at finding relations such as ***A** is to **B** as **C** is to* __. For instance, in the relation ***good** is to **better** as **bad** is to* __, the correct answer would be ***worse***.

the model given the learned parameters before computing the similarities to the other patents.

A2 Functions for measuring similarity between text documents

Transforming the patent documents into numeric feature vectors allows to assess their similarity with the help of mathematical functions. Rieck and Laskov [12] give a comprehensive overview on vectorial similarity measures for the pairwise comparison of sequential data. These can be divided into three main categories, namely *kernels*, *distance functions*, and *similarity coefficients*. Their formulas are shown in Table A and the notation is consistent with the one in the paper. Here, w corresponds to a word in the vocabulary L of the corpus, and $\Phi_w(x)$ maps each word $w \in L$ to its normalized and weighted count in sequence x, i.e. to its *tf-idf* value. The similarity functions will be briefly described in the following, while further details can be found in the original publication [12]. The general idea for the comparison of two sequences is that the more

Table A. Overview of similarity measures for sequential data [12].

Similarity coefficients	
Cosine	$\frac{\sum_{w\in L}\Phi_w(x_i)\Phi_w(x_j)}{\sqrt{\sum_w \Phi_w(x_i)^2}\sqrt{\sum_w \Phi_w(x_j)^2}}$
Braun-Blanquet	$\frac{a}{\max(a+b,a+c)}$
Czekanowski, Sørensen-Dice	$\frac{2a}{(2a+b+c)}$
Jaccard	$\frac{a}{(a+b+c)}$
Kulczynski	$\frac{a}{2(a+b)}+\frac{a}{2(a+c)}$
Otsuka, Ochiai	$\frac{a}{\sqrt{(a+b)(a+c)}}$
Simpson	$\frac{a}{\min(a+b,a+c)}$
Sokal-Sneath, Anderberg	$\frac{a}{(a+2(b+c))}$
**Kernel functions**	
Linear	$\sum_{w\in L}\Phi_w(x_i)\Phi_w(x_j)$
Gaussian	$\exp\left(\frac{-d(x_i,x_j)^2}{2\sigma^2}\right)$
Histogram intersection	$\sum_{w\in L}\min\left(\Phi_w(x_i),\Phi_w(x_j)\right)$
Polynomial	$\left(\sum_{w\in L}\Phi_w(x_i)\Phi_w(x_j)+\Theta\right)^p$
Sigmoidal	$\tanh\left(\sum_{w\in L}\Phi_w(x_i)\Phi_w(x_j)+\Theta\right)$
**Distance functions**	
Canberra	$\sum_{w\in L}\frac{\lvert\Phi_w(x_i)-\Phi_w(x_j)\rvert}{\Phi_w(x_i)+\Phi_w(x_j)}$
Chebyshev	$\max_{w\in L}\lvert\Phi_w(x_i)-\Phi_w(x_j)\rvert$
Euclidean	$\sum_{w\in L}\lvert\Phi_w(x_i)-\Phi_w(x_j)\rvert^2$
Geodesic	$\arccos\sum_{w\in L}\Phi_w(x_i)\Phi_w(x_j)$
Hellinger^2	$\sum_{w\in L}\left(\sqrt{\Phi_w(x_i)}-\sqrt{\Phi_w(x_j)}\right)^2$
Jensen-Shannon	$\sum_{w\in L}H\left(\Phi_w(x_i),\Phi_w(x_j)\right)$
Manhattan	$\sum_{w\in L}\lvert\Phi_w(x_i)-\Phi_w(x_j)\rvert$
Minkowski^p	$\sum_{w\in L}\lvert\Phi_w(x_i)-\Phi_w(x_j)\rvert^p$
χ^2	$\sum_{w\in L}\frac{(\Phi_w(x_i)-\Phi_w(x_j))^2}{\Phi_w(x_i)+\Phi_w(x_j)}$

overlap they show with respect to their subsequences, the more similar they are. When transforming texts into BOW features, a subsequence corresponds to a single word. Two sequences x_i and x_j can thus be compared based on the normalized and weighted counts of the subsequences stored in the respective feature vectors $\mathbf{x}_i$ and $\mathbf{x}_j$.

Kernel functions The first group of similarity measures Rieck and Laskov [12] discuss are kernel functions. They implicitly map the feature vectors into a possibly high or even infinite dimensional feature space, where the kernel can be expressed as a dot product. A kernel k thus has the general form

$$k\left(\mathbf{x}_i, \mathbf{x}_j\right) = \langle f(\mathbf{x}_i), f(\mathbf{x}_j)\rangle,$$

where f maps the vectors into the kernel feature space. The advantage of the kernel function is that it avoids the explicit calculation of the vectors' high dimensional mapping and allows to obtain the result in terms of the vectors' representation in the input space instead [13, 14].

Distance functions The distance functions described in Rieck and Laskov [12] are so-called *bin-to-bin* distances [15]. This means that they compare each component of the vector to its corresponding component in the other one, e.g. by subtracting the respective word counts and summing the subtractions for all words in the vocabulary. Unlike similarity measures, the distance measures are higher the more different the compared sequences are but can be easily transformed into a similarity measure by multiplying the result with -1, for example.

Similarity coefficients Similarity coefficients were designed for the comparison of binary vectors and, instead of expressing metric properties, they assess similarity by comparing the number of matching components between two sequences. More precisely, for calculating the similarity of two sequences x_i and x_j, they use three variables a, b and c, where a corresponds to the number of components contained in both x_i and x_j, b to the number of components contained in x_i but not in x_j, and c to the number of components contained in x_j but not in x_i. In the case of BOW vectors, which are not inherently binary, the three variables can be expressed as follows:

$$\begin{aligned}
a &= \sum_{w \in L} \min\left(\Phi_w(x_i), \Phi_w(x_j)\right), \\
b &= \sum_{w \in L} \left(\Phi_w(x_i) - \min\left(\Phi_w(x_i), \Phi_w(x_j)\right)\right), \\
c &= \sum_{w \in L} \left(\Phi_w(x_j) - \min\left(\Phi_w(x_i), \Phi_w(x_j)\right)\right).
\end{aligned}$$

Supporting information B: Data

Patent corpus

To evaluate the different methods for computing document similarities on real world data, an initial patent corpus was obtained from a patent database. This corpus consists of over 100,000 patent grants and applications published at the *United States Patent and Trademark Office* (USTPO) between 2000 and 2015.

We create such a patent corpus (by crawling GOOGLE PATENTS[2]) as illustrated in Fig A. To get a more homogeneous dataset, only patents of the category A61 (*medical or veterinary science and hygiene*) according to the *Cooperative Patent Classification scheme* (CPC) were included in our corpus. Another important criterion for including a patent document in our initial patent corpus was that its search report, i.e. the prior art cited by the examiner, had to be available from the database. Starting with 20 manually selected (randomly chosen) seed patents published in 2015, the patent corpus was iteratively extended by including the seed patents' citations if they were published after 1999 and belonged to the category A61. The citations of these patents were then again checked for publication year and category and included if they fulfilled the respective conditions.

STRUCTURE OF THE CRAWLED DATASET

Comparing the distribution of patents published per year in the dataset and the total amount of patents filed between 2000 and 2015 at the USTPO (Fig B), it can be seen that the distribution in the dataset is not representative. The peak in 2003 and the fact that there are less and less patents with a publication date in the following years is most probably a result of the crawling strategy. Given that we started with some patents filed in 2015 and then subsequently crawled the citations, published in the past, explains the low amount of patents published in more recent years in the dataset.

[2] https://www.google.de/patents

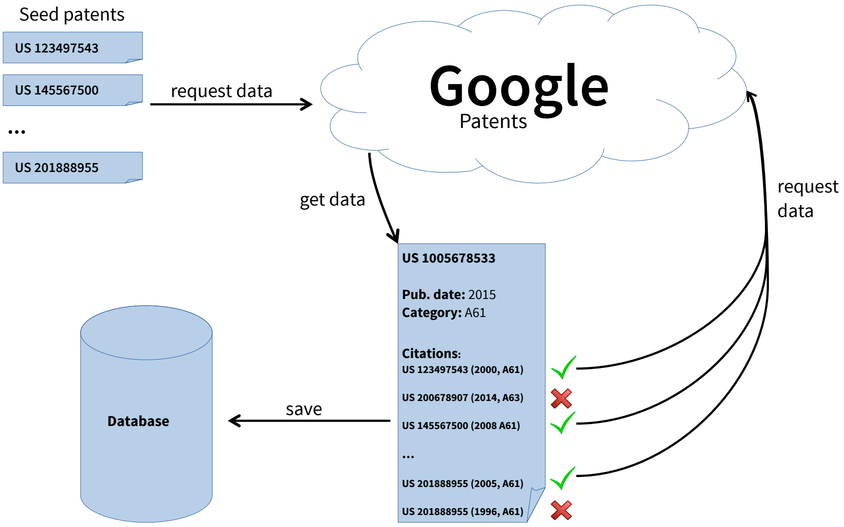

Fig A. Illustration of the crawling process.

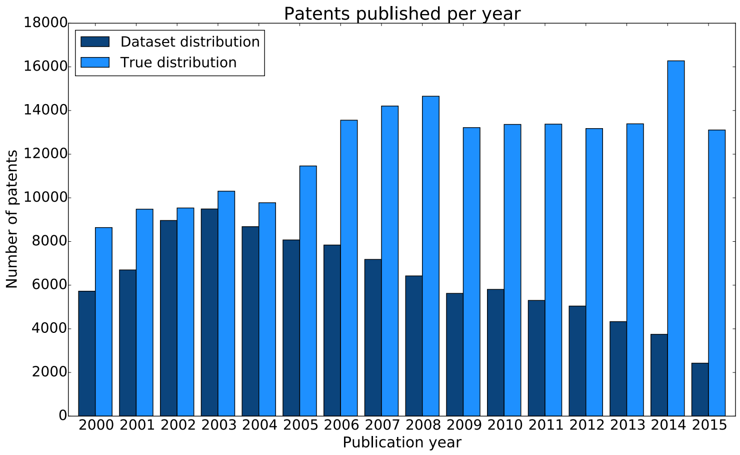

Fig B. Number of published patents per year in the crawled dataset compared to the true distribution of patents published per year.

The same holds for the subcategory distribution displayed in Fig C. While the most prominent subcategory in our dataset is A61B, the most frequent subcategory is actually A61K. The bias for subcategory A61B is due to the fact that several seed patents belonged to it.

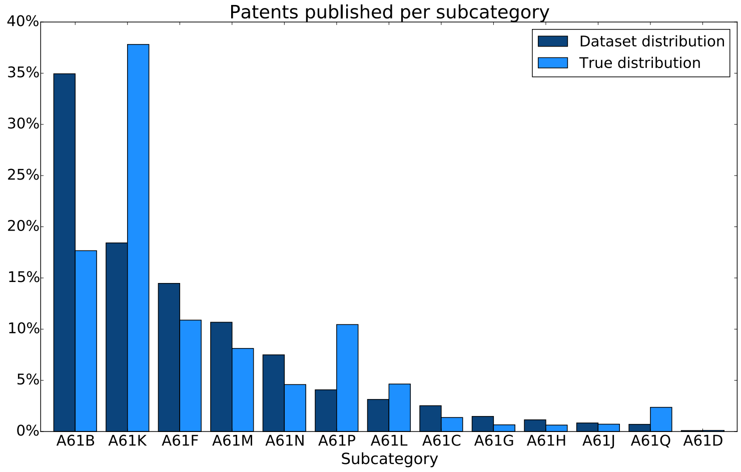

Fig C. Subcategory distribution for crawled and published patents from 2000 to 2015. Subcategory names can be found in Table B.

Finally, to get some insights into the existing search for prior art, we examine the distribution of the number of citations in the patent dataset. The citation counts for a subsample of 5000 randomly selected patents show that the distribution follows

Table B. CPC table for the subcategories of class **A61** (MEDICAL OR VETERINARY SCIENCE AND HYGIENE).

**A61B**	Diagnosis<br>Surgery<br>Identification
**A61C**	Dentistry<br>Apparatus or methods for oral or dental hygiene
**A61D**	Veterinary instruments, implements, tools, or methods
**A61F**	Filters implantable into blood vessels<br>Prostheses<br>Devices providing patency to or preventing collapsing of tubular structures of the body, e.g. stents<br>Orthopaedic, nursing or contraceptive devices<br>Fomentation<br>Treatment or protection of eyes or ears<br>Bandages, dressings or absorbent pads<br>First-aid kits
**A61G**	Transport or accomodation for patients<br>Operating tables or chairs<br>Chairs for dentistry<br>Funeral devices
**A61H**	Physical therapy apparatus, e.g. devices for locating or stimulating reflex points in the body<br>Artificial respiration<br>Massage<br>Bathing devices for special therapeutic or hygienic purposes or specific parts of the body
**A61J**	Containers specially adapted for medical or pharmaceutical purposes<br>Devices or methods specially adapted for bringing pharmaceutical products into particular physical or administering forms<br>Devices for administering food or medicines orally<br>Baby comforters<br>Devices for receiving spittle
**A61K**	Preparations for medical, dental, or toilet purposes
**A61L**	Methods or apparatus for sterilising materials or objects in general<br>Disinfection, sterilisation, or deodorisation of air<br>Chemical aspects of bandages, dressings, absorbent pads, or surgical articles<br>Materials for bandages, dressings, absorbent pads, or surgical articles
**A61M**	Devices for introducing media into, or onto, the body<br>Devices for transducing body media or for taking media from the body<br>Devices for producing or ending sleep or stupor
**A61N**	Electrotherapy<br>Magnetotherapy<br>Radiation therapy<br>Ultrasound therapy
**A61Q**	Specific use of cosmetics or similar toilet preparations

Zipf's law with many patents having very few citations and a low number of patents having many citations (Fig D).

STRUCTURE OF A PATENT

The requirements regarding the structure of a patent application are very strict and prescribe the presence of certain sections and what their content should be. For the automated comparison of texts it can be interesting to have a closer look at the different sections of the documents as it might, for instance, be sufficient to only compare a specific section of the texts. This can on the one hand be useful to perform a preliminary search for prior art before the patent text is written in its entirety in order to prevent unnecessary work and on the other hand, it can help to decrease the computational burden of preprocessing and comparing full texts.

The *Patent Cooperation Treaty* (PCT) by the *World Intellectual Property Organization* (WIPO) defines several obligatory sections a patent application must contain.[3] According to their requirements, a patent application should consist of a title, an abstract, the claims, and the description, where the invention is thoroughly described and the figures included in the document are explained in depth. Similar to scientific publications, a patent's abstract consists of a short summary of what the invention is about. The claims section plays a very special role in a patent application, as it defines the extent of the

[3]The WIPO is an agency of the *United Nations* with the aim of unifying and fostering the protection of intellectual property.

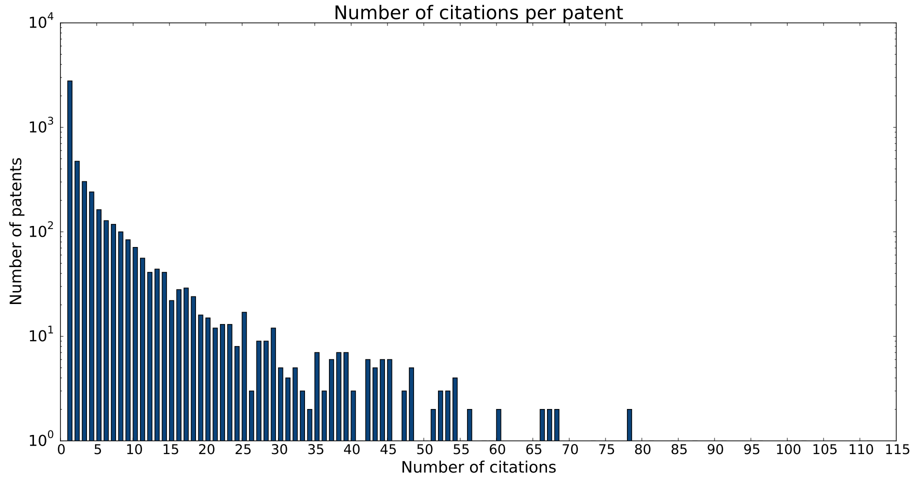

Fig D. Number of patents on a logarithmic scale per number of citations for a subsample of 5000 randomly selected patents.

protection the patent should guarantee for the invention and is therefore the section the patent attorneys and patent officers base their search for prior art on. If the claims enter in conflict with already existing publications, they can be edited by weakening the protection requirements, which is why this section is reformulated the most during the possibly multiple stages of a patent process.

As both the USTPO and the *European Patent Office* (EPO) adopt the PCT, the required sections are the same in the United States and in Europe. Nonetheless, some differences in the length of the description section can be observed. For a patent application handed in at the USTPO, this section mostly consists of the figures' descriptions, while for applications to the EPO it contains more abstract descriptions of the invention itself. This is due to stricter requirements of consistency between claims and description for European patents and must be taken into account when patents filed at different offices are compared, as this might result in lower similarity scores [16, 17].

Constructing a labelled dataset with cited and random patents

A first labelled dataset was constructed from the patent corpus by pairing up the patents and labelling each pair depending on whether or not one patent in the pair is cited by the other. More formally, let $\mathcal{P}$ be the set of patents in the corpus and $\mathcal{P}^2$ its Cartesian product. Each patent pair $(p_1, p_2) \in \mathcal{P}^2$ then gets assigned the label 1 (*cited*) if p_2 is contained in the search report of patent p_1 and 0 (*random*) otherwise. As some of the tested approaches are computationally expensive, we did not pair up all of the 100,000 documents in the corpus. Instead, the roughly 2,500 patents published in 2015 contained in the corpus were selected as a set of target patents and paired up with their respective citations as well as with a set of 1,000 randomly selected patents that were not contained in the search reports of any of the target patents.

Due to divisional applications and parallel filings and because claims are often changed during the application process, patents with the same description may appear several times with different IDs, which is why, as a sanity check, duplicates for some of the target patents were included in the dataset as well.[4] All together, this 'cited/random' labelled dataset consists of 2,470,736 patent pairs, of which 41,762 have a citation, 2,427,000 a random, and 1974 a duplicate relation.

Obtaining relevancy labels from a patent attorney

As a subsample of the first dataset, our second dataset was constructed by taking ten of the target patents published in 2015, as well as their respective cited patents. In addition to that, in order to assess if relevant patents were missing from the search report, some of the random patents were included as well. These were selected based on their cosine similarity to the target patent, computed using the BOW vector representations. We chose for each patent the ten highest-scored, ten very low-ranked, and ten mid-ranked random patents. In total, this dataset subsample consists of 450 patent pairs, of which 151 are citations and 299 random pairs.

Neither knowing the similarity score of the patent pairs nor which ones were cited or random patents, the patent attorney manually assigned a score between 0 and 5 to the patent pairs according to how relevant the respective document was considered for the target patent, thus yielding the second labelled dataset. For most of the following evaluation, the patent attorney's scoring was transformed into a binary labelling by considering all patent pairs with a score greater than 2 as relevant and the others as irrelevant.

[4]Duplicates are expected to receive a similarity score near or equal to 1.

Supporting information C: Evaluation

Computing AUC scores to evaluate similarity measures

When computing similarity scores for all patent pairs, this results in two distributions of similarity scores, one for the positive samples (pairs of patents where one patent was cited by the other) and one for the negative samples (random patents). Ideally, these two distributions of scores would be separated, such that it is easy to chose a threshold to identify a positive or negative sample based on the corresponding similarity score of the patent pair (Fig E). To measure how well these two distributions are separated, we can compute the area under the *receiver operating characteristic* (ROC) curve. Every possible threshold value chosen for separating positive from negative examples can lead to some pairs of unrelated patents to be mistakenly considered as relevant, what is called *false positives* (FP), or to pairs of related patents mistakenly regarded as irrelevant, so-called *false negatives* (FN). Correct decisions are either *true negatives* (TN), i.e., a pair of random patents that was correctly considered as irrelevant, or *true positives* (TP), which are correctly detected cited patents. Based on this, for every threshold value we can compute the *true positive rate* (TPR), also called *recall*, the *false positive rate* (FPR), and the *false negative rate* (FNR) to set wrong and correct decisions into relation:

$$TPR = \frac{TP}{TP + FN},$$
$$FPR = \frac{FP}{FP + TN},$$
$$FNR = \frac{FN}{TP + FN}.$$

By plotting the TPR against the FPR for different decision similarity score thresholds, we then obtain the graph of the ROC curve, where the *area under the* ROC *curve* (AUC) conveniently translates the performance of the similarity measure into a number between 0.5 (no separation between distributions) and 1 (clear distinction between positive and negative samples), as shown in Fig E.[5]

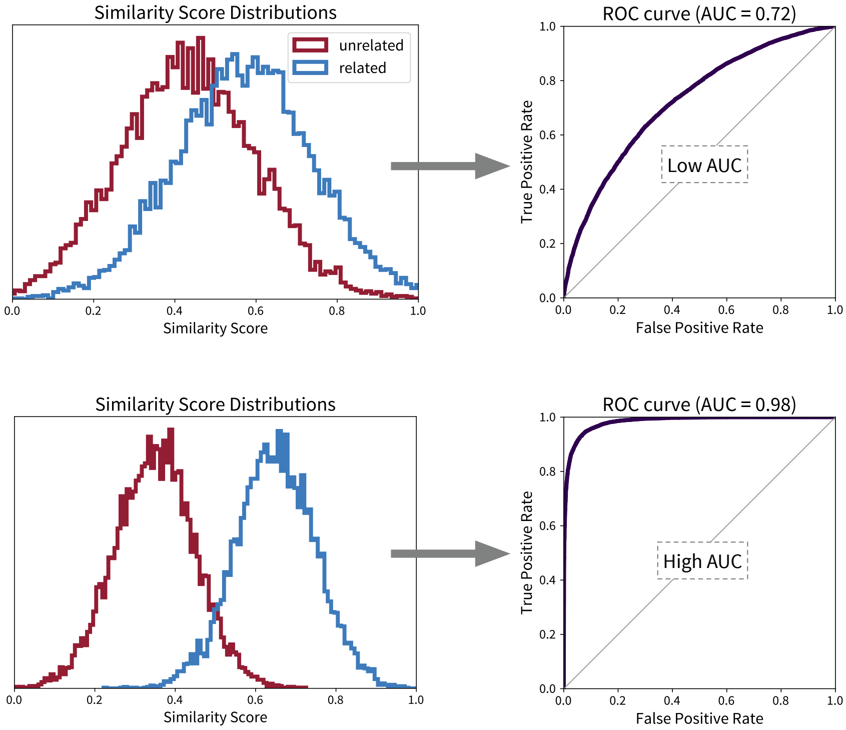

Fig E. ROC curve and AUC derived from similarity score distributions. Similarity scores were generated using artificial data to show how the difference in similarity scores for related *(blue)* and unrelated sample pairs *(red)* influence the ROC curves and therefore result in a lower or higher AUC.

[5]Many information retrieval applications use precision and recall to measure the system's performance by comparing the number of relevant documents to the number of retrieved documents. However, since we do not only want to retrieve relevant documents, but in general select a discriminatory, interpretable, and meaningful similarity score, we consider the AUC, which relates the system's recall to its FPR.

Supporting information D: Results

D1 Identifying cited patents using different similarity functions with BOW features

We evaluated all similarity measures listed in Table A using BOW features on the cited/random corpus. When computing the BOW features, we either used the term frequency (*tf*) or a binary flag (0/1) for each word occurring in a document and experimented with raw values as well as values weighted by the words' *idf* scores. Furthermore, these feature vectors were either normalized by the vector's maximum value or its length. The AUC scores for all these combinations can be found in Table C.

For all similarity functions (excluding the Minkowski distance) the best result is obtained when using either *tf* (distance functions) or *tf-idf* (kernel functions, similarity coefficients, as well as Canberra and Euclidean distance) feature vectors. This shows that it is important to consider how often each term occurs in the documents instead of only encoding its presence or absence. Another observation that can be made is that the majority of the highest AUC scores is obtained on the *tf-idf* feature vectors, which give a more accurate insight on how important each term actually is for the given document and reduce the importance of *stop words*. Except for the Chebychev distance, the final normalization of the vectors should be performed using their lengths and not their maximum values. This might be due to the fact that the length normalization takes all the vector entries into account and not only the highest one, which makes it less sensitive to outliers, i.e. extremely high values in the vector. With length normalized vectors as input, the linear kernel is equal to the cosine similarity and can thus be included into the group of similarity coefficients.

All in all, except for the Euclidean distance, which gives the same AUC as the cosine similarity using normalized vectors, the kernel functions and similarity coefficients yield much better results than the distance measures, which shows that it is more important to focus on words the texts have in common instead of calculating their distance in the vector space. Among similarity coefficients and kernel functions, the former function class gives slightly more robust results. Given that similarity coefficients are especially designed for sequence comparison by explicitly taking into account their subsequences' overlap, they seem to be the appropriate function class for measuring similarity between the BOW feature vectors.

The cosine similarity is widely used in information retrieval [18–20] and is well suited to distinguish between cited and random patents as it assigns lower scores to random than to cited patent pairs and, additionally, reliably detects duplicates by assigning them a score near or equal to 1 (Fig 2 in the main paper).

D2 Detailed examination of outliers in the citation process

For a better understanding of the disagreements between the cited/random labelling and the cosine similarity scores compared to the relevant/irrelevant labelling, we take a closer look at a FP yielded by the cosine similarity as well as a FP yielded by both, the cosine similarity and the cited/random labelling. In addition to that, in the main text we gave an example of a FN, i.e. a relevant patent that was missed by the patent examiner, but would have been found by our automated approach, as it received a high similarity score.

False positive yielded by our automated approach The patent with ID US7585299[6] marked with a gray circle in Fig F on the left would correspond to a FP taking both human labellings as the ground truth, because it received a high cosine similarity score although being neither relevant nor a citation. The target patent (ID US20150066086[7]) as well as the patent with ID US7585299 describe inventions that stabilize vertebrae. In the target patent, the described device clamps adjacent spinous processes together by two plates held together by two screws without introducing screws inside the bones. The device described in patent US7585299, in contrast, stabilizes the spine using bone anchors, which are screwed e.g. into the spinous processes or another part of the respective vertebrae and which have a clamp on the opposite end. The vocabulary in both patents is thus extremely similar, which leads to a high overlap on the BOW vector level, however, the two devices are far too different to be considered as similar inventions given that one is rigid and screwed into the bones whereas the other one only clamps the spinous processes and thereby guarantees a certain degree of flexibility.

False positive yielded by our automated approach and the cited/random labelling For other target patents, more discordance with respect to the relevance of the other patents can be observed, also between the two human ratings. The correlation of the relevant/irrelevant scoring for the patent with ID US20150066087[8] in Fig F on the right shows that there are many cited patents that received a rather low score by the patent attorney, which means that the patent examiner produced a considerable amount of FP. One possible explanation for this might be that the patent examiners tend to produce rather more than less citations and thus include a large amount of the patents that are returned as results for their keyword query

[6] http://www.google.de/patents/US7585299
[7] http://www.google.de/patents/US20150066086
[8] http://www.google.de/patents/US20150066087

Table C. AUC scores for all the tested combinations of BOW feature extraction approaches and similarity functions on the cited/random corpus.

Similarity coefficients

	normalization	*tf*	0/1	*tf-idf*	0/1-*idf*
Braun-Blanquet	length	0.8550	0.7941	**0.9480**	0.8791
	max	0.8075	0.7941	0.9338	0.8753
Czekanowski, Sørensen-Dice	length	0.8749	0.8371	**0.9555**	0.9021
	max	0.8593	0.8680	0.9505	0.9144
Jaccard	length	0.8749	0.8371	**0.9555**	0.9021
	max	0.8593	0.8680	0.9505	0.9144
Kulczynski	length	0.8767	0.8536	**0.9574**	0.9122
	max	0.8761	0.9079	0.9571	0.9266
Otsuka, Ochiai	length	0.8759	0.8451	**0.9568**	0.9072
	max	0.8687	0.8982	0.9558	0.9323
Simpson	length	0.8566	0.8982	**0.9543**	0.9268
	max	0.8190	0.7879	0.9479	0.8685
Sokal-Sneath, Anderberg	length	0.8749	0.8371	**0.9555**	0.9021
	max	0.8593	0.8680	0.9505	0.9144

Kernel functions

	normalization	*tf*	0/1	*tf-idf*	0/1-*idf*
Linear	length*+	0.7336	0.8982	**0.9560**	0.9470
	max	0.5411	0.7142	0.9387	0.8168
Gaussian	length	0.7336	0.8982	**0.9560**	0.9470
	max	0.6909	0.5010	0.6366	0.5083
Histogram intersection	length	0.7853	0.8050	**0.9239**	0.8759
	max	0.6939	0.7142	0.8969	0.7694
Polynomial	length	0.7336	0.8982	**0.9480**	0.9468
	max	0.5411	0.7142	0.9383	0.8168
Sigmoidal	length	0.7336	0.8982	**0.9560**	0.9470
	max	0.5411	0.5000	0.9387	0.7971

Distance functions

	normalization	*tf*	0/1	*tf-idf*	0/1-*idf*
Canberra	length	0.5253	0.5479	**0.6523**	0.6184
	max	0.5259	0.5937	0.6072	0.5438
Chebyshev	length	0.6252	0.5686	0.6056	0.5473
	max	**0.6271**	0.5000	0.6162	0.5006
Hellinger	length	**0.8746**	0.6709	0.7213	0.6183
	max	0.8064	0.5937	0.6559	0.5788
Jensen-Shannon	length	**0.8607**	0.6699	0.7028	0.6173
	max	0.7889	0.5937	0.6415	0.5787
Manhattan	length	**0.7987**	0.6486	0.6437	0.6002
	max	0.7239	0.5937	0.5997	0.5767
Minkowski ($p = 3$)	length	0.6765	**0.7203**	0.6934	0.5930
	max	0.6606	0.5937	0.6616	0.5846
Euclidean	length+	0.7336	0.8982	**0.9560**	0.9470
	max	0.6909	0.5937	0.6366	0.5794
χ^2	length	**0.8476**	0.6686	0.7567	0.6134
	max	0.7739	0.5937	0.6180	0.5543

The best result for each similarity function is printed in bold and the best result for each function class is underlined.

* The linear kernel with length normalized vectors corresponds to the cosine similarity.

+ The AUC is equal, as for length normalized vectors (i.e. $\|\mathbf{x}_i\|_2 = \|\mathbf{x}_j\|_2 = 1$), we get $\|\mathbf{x}_i - \mathbf{x}_j\|_2^2 = (\mathbf{x}_i - \mathbf{x}_j)^T(\mathbf{x}_i - \mathbf{x}_j) = \mathbf{x}_i^T\mathbf{x}_i - 2\mathbf{x}_i^T\mathbf{x}_j + \mathbf{x}_j^T\mathbf{x}_j = 1 - 2\mathbf{x}_i^T\mathbf{x}_j + 1 = 2 - 2\mathbf{x}_i^T\mathbf{x}_j$ and $\mathbf{x}_i^T\mathbf{x}_j$ is equal to the cosine similarity.

into the search report, although, on closer inspection, the relevance for the target patent is unfounded. This is also due to the fact that they mostly base their search on the claims section, which is usually kept as general as possible to guarantee a maximum degree of protection for the invention. The analysis of the FP with ID US20130079880[9] (marked by the gray circle in the plot) underpins this hypothesis. The claims sections of the two patents are similar and the devices described in the patents are of similar construction, both having plates referred to as wings. The device described in the target patent,

[9] http://www.google.de/patents/US20130079880

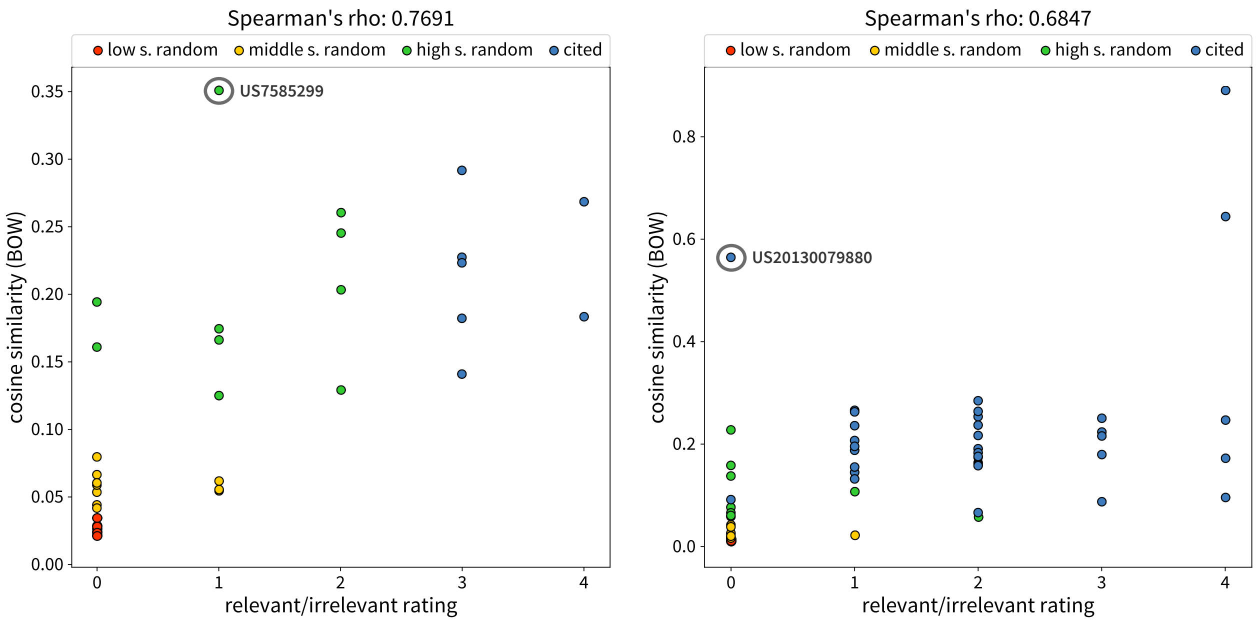

Fig F. False positives. *Left:* Score correlation for the patent with ID US20150066086 with a false positive (ID US7585299) yielded by the cosine similarity, circled in gray. *Right:* Score correlation for the patent with ID US20150066087 with a false positive (ID US20130079880) yielded by the cosine similarity and the cited/random labelling circled in gray. The blue dots correspond to the cited and the green, yellow, and red dots to the random patents whose colors describe whether they received a high, middle, or low cosine similarity score.

however, is designated to immobilize adjacent spinous processes, whereas the one described in patent US20130079880 is aimed at increasing the space between two adjacent vertebrae to relieve pressure caused for instance by dislocated discs. Especially the similar claims section might have led the patent examiner to cite the patent, although the devices clearly have different purposes, which can easily be derived from their descriptions.

References

[1] Manning CD, Raghavan P, Schütze H. Scoring, term weighting and the vector space model. In: Introduction to Information Retrieval. Cambridge University Press; 2008.

[2] Landauer TK, Foltz PW, Laham D. An introduction to latent semantic analysis. Discourse processes. 1998;25:259–284.

[3] Schölkopf B, Smola A, Müller KR. Nonlinear Component Analysis as a Kernel Eigenvalue Problem. Neural Computation. 1998;10(5):1299–1319.

[4] Müller KR, Mika S, Rätsch G, Tsuda K, Schölkopf B. An introduction to kernel-based learning algorithms. IEEE Transactions on Neural Networks. 2001;12(2):181–201.

[5] Zhang L. PatSearch: An Integrated Framework for Patent Document Retrieval. In: An Integrated Framework for Patent Analysis and Mining. FIU Electronic Theses and Dissertations; 2016.

[6] Bengio Y, Ducharme R, Vincent P, Janvin C. A Neural Probabilistic Language Model. J Mach Learn Res. 2003;3:1137–1155.

[7] Mikolov T, Chen K, Corrado G, Dean J. Efficient estimation of word representations in vector space. arXiv preprint arXiv:13013781. 2013;.

[8] Mikolov T, Sutskever I, Chen K, Corrado G, Dean J. Distributed Representations of Words and Phrases and their Compositionality. Advances in Neural Information Processing Systems. 2013; p. 3111–3119.

[9] Horn F. Context encoders as a simple but powerful extension of word2vec. In: Proceedings of the 2nd Workshop on Representation Learning for NLP. Association for Computational Linguistics; 2017. p. 10–14.

[10] Mikolov T, Yih Wt, Zweig G. Linguistic Regularities in Continuous Space Word Representations. In: Proceedings of the 2013 Conference of the North American Chapter of the Association for Computational Linguistics: Human Language Technologies; 2013. p. 746–751.

[11] Le Q, Mikolov T. Distributed Representations of Sentences and Documents. In: Jebara T, Xing EP, editors. Proceedings of the 31st International Conference on Machine Learning (ICML-14). JMLR Workshop and Conference Proceedings; 2014. p. 1188–1196.

[12] Rieck K, Laskov P. Linear-Time Computation of Similarity Measures for Sequential Data. J Mach Learn Res. 2008;9:23–48.

[13] Schölkopf B, Smola AJ. A Short Introduction to Learning with Kernels. In: Mendelson S, Smola AJ, editors. Advanced Lectures on Machine Learning: Machine Learning Summer School 2002 Canberra, Australia, February 11–22, 2002 Revised Lectures. Berlin, Heidelberg: Springer Berlin Heidelberg; 2003. p. 41–64.

[14] Schölkopf B, Smola AJ. Learning with kernels: support vector machines, regularization, optimization, and beyond. MIT press; 2002.

[15] Pele O. Distance Functions: Theory, Algorithms and Applications. The Hebrew University of Jerusalem; 2011.

[16] Piroi F, Hanbury A. Evaluating Information Retrieval Systems on European Patent Data: The CLEF-IP Campaign. In: Lupu M, Mayer K, Kando N, Trippe AJ, editors. Current Challenges in Patent Information Retrieval. Berlin, Heidelberg: Springer Berlin Heidelberg; 2017. p. 113–142.

[17] Lupu M, Fujii A, Oard DW, Iwayama M, Kando N. Patent-Related Tasks at NTCIR. In: Lupu M, Mayer K, Kando N, Trippe AJ, editors. Current Challenges in Patent Information Retrieval. Berlin, Heidelberg: Springer Berlin Heidelberg; 2017. p. 77–111.

[18] Crocetti G. Textual Spatial Cosine Similarity. CoRR. 2015;abs/1505.03934.

[19] Huang A. Similarity measures for text document clustering. In: Proceedings of the Sixth New Zealand Computer Science Research Student Conference (NZCSRSC2008); 2008. p. 49–56.

[20] Baeza-Yates RA, Ribeiro-Neto B. Modeling. In: Modern Information Retrieval. Boston, MA, USA: Addison-Wesley Longman Publishing Co., Inc.; 1999.
